# A Rare Infectious Complication of a Treatment With Biologics: Disseminated Cutaneous Leishmaniasis Associated With Adalimumab

**DOI:** 10.5826/dpc.1003a61

**Published:** 2020-06-29

**Authors:** Kerasia-Maria Plachouri, Maria Gkermpesi, Eleftheria Vryzaki, Pinelopi Kollyrou, Markos Marangos, Sophia Georgiou

**Affiliations:** 1 Department of Dermatology, University General Hospital of Patras, Greece; 2 Department of Pathology, University General Hospital of Patras, Greece; 3 Division of Infectious Diseases, University General Hospital of Patras, Greece

**Keywords:** adalimumab, leishmaniasis, immunosuppression, psoriasis, biologics

## Introduction

Leishmaniasis is caused by an intracellular protozoon and is characterized by a broad spectrum of cutaneous, mucosal, and systemic presentations [1]. The clinical manifestations of leishmaniasis are determined by the subtype of the infectious agent and the immunocompetent profile of the patient [1]. The introduction of biologics is accompanied with impressive therapeutic outcomes, but also with an increase in the incidence of several infectious diseases [1]. We present the case of a patient who developed a disseminated cutaneous leishmaniasis infection during treatment with adalimumab.

## Case Presentation

**Figure 1:**
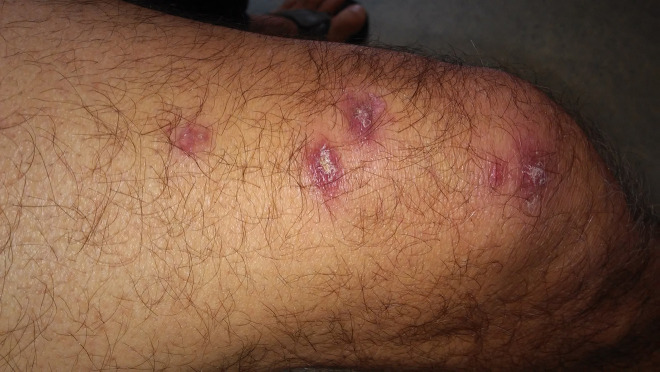
Cutaneous leishmaniasis lesions caused by *L infantum* on the left thigh of the patient.

A 68-year-old man from southwestern Greece presented in our dermatology department because of the appearance of 10 disseminated, centrally erosive, erythematous cutaneous nodules and plaques, approximately 1.5 to 2 cm in diameter, distributed in the trunk and the lower extremities, partially in a sporotrichoid pattern, which had been persistent over 7 months prior to the referral (Figure 1). The patient was a farmer and had been receiving ongoing treatment with methotrexate (7.5 mg subcutaneously every week) and adalimumab (40 mg subcutaneously every other week) over a period of 9 years for plaque psoriasis and psoriatic arthritis. At the time of presentation both diseases were in long-term remission, with no signs of cutaneous psoriatic lesions, lymphadenopathy, or arthritis during the physical examination.

**Figure 2:**
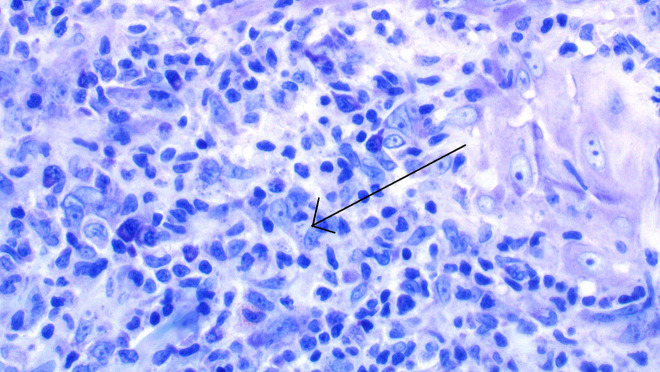
Histopathological image of cutaneous leishmaniasis. Dense dermal infiltrate of large histiocytes. Leishman-Donovan bodies are found in histiocytes, lining their internal membrane (arrow). An admixture of lymphocytes, neutrophils, and plasma cells are also present (arrow) (Giemsa stain, ×400).

**Figure 3:**
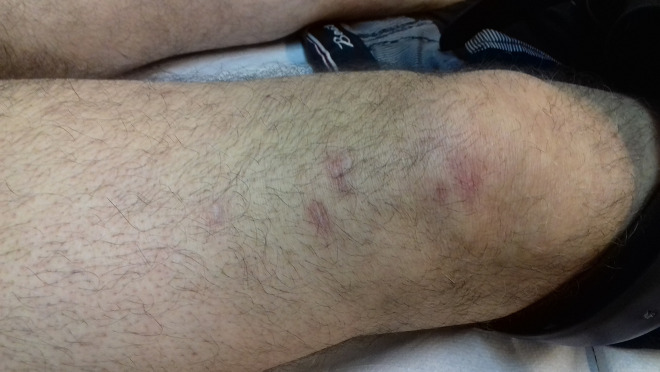
Almost complete clearance of the leishmaniasis lesions on the left thigh of the patient (24-month follow-up).

The histopathological examination of one of the skin lesions showed evidence of a *Leishmania* spp infection (Figure 2). The agent was identified as *L infantum*, using the polymerase chain reaction (PCR) technique in a skin biopsy sample. The screening for visceral leishmaniasis, which included the measurement of antibodies against the recombinant k39 antigen, the specific anti-Leishmania antibody titers in blood serum, as well as PCR in a bone marrow aspirate sample, showed no Leishmania-associated findings. An ultrasound examination showed no signs of hepatosplenomegaly and a chest x-ray revealed no abnormal findings. Owing to the occurrence of 10 or more disseminated lesions in more than 2 noncontinuous anatomic locations, the condition was characterized as a disseminated cutaneous leishmaniasis. The treatment with adalimumab and methotrexate was interrupted and treatment with liposomal amphotericin B was initiated (3 mg/kg/day, administered intravenously on days 1–5, 10, 17, 24, 31, and 38). The therapy was well tolerated. In the 24-month follow-up the patient remained asymptomatic, with almost total clearance of the cutaneous lesions (Figure 3). Adalimumab was reinitiated 1.5 years after the successful eradication of leishmaniasis, with no signs of recurrence over the 48-month follow-up (Figure 3).

## Conclusions

In the era of biologics, physicians often witness the reactivation of latent infections, such as tuberculosis or leishmaniasis [1]. When it comes to leishmaniasis in relation to tumor necrosis factor alpha (TNF-α) inhibitors, one of the suggested pathogenetic mechanisms of infection is believed to be a TNF-α inhibitor–mediated downregulation of endothelial adhesion molecules such as E-selectin and intercellular adhesion molecule 1 [1]. The latter are necessary for the mononuclear cell recruitment and the formation of granulomas that enable the eradication of *Leishmania* spp [1].

Forty-nine cases of anti-TNF-α–associated leishmaniasis are published in the literature, 28 with cutaneous leishmaniasis, 16 with visceral leishmaniasis, and 5 with mucocutaneous leishmaniasis [1]. All patients with visceral leishmaniasis and mucocutaneous leishmaniasis received a systemic treatment; the therapy of choice was in most cases intravenous liposomal amphotericin B in various dosage regimens with a cumulative dose ranging from 15.4 mg/kg to 50 mg/kg, while less frequently parenteral pentavalent antimonials and miltefosine were also administered [1]. Half of the patients with cutaneous leishmaniasis received a systemic treatment with liposomal amphotericin B as well, while the rest of the cases were treated with intralesional pentavalent antimonials, occasionally combined with surgical excision or cryotherapy [1]. It is interesting that the TNF-α inhibitor treatment had to be stopped in only 32 of the reported cases [1]. All patients showed a full recovery, but relapses were reported in 3 patients treated with intralesional pentavalent antimonials, 1 patient treated with surgery, and only 2 patients treated with systemic liposomal amphotericin B and miltefosine, respectively [1].

Although these data seem relevant only for areas where these infectious diseases are endemic, such as South Europe and South America, one cannot underestimate the fact that the massive human migration for socioeconomic reasons may gradually change the epidemiological and demographic aspects of infectious diseases [2]. It is necessary for physicians to gain familiarity with leishmaniasis and to maintain a high level of suspicion, especially in iatrogenically immunosuppressed patients, in order to reach an early diagnosis and thus prevent disease-related complications as well as disease transmission in nonendemic areas [2].
